# The Association Between Self‐Perceptions of Ageing and Foot and Lower‐Limb Health in Community‐Dwelling Older Adults

**DOI:** 10.1002/jfa2.70172

**Published:** 2026-06-08

**Authors:** Prue Molyneux, Ella Bloomfield, Miles Broderick, Sarah Stewart

**Affiliations:** ^1^ School of Allied Health Auckland University of Technology Auckland New Zealand; ^2^ AUT Active Living and Rehabilitation Research Centre School of Allied Health Auckland University of Technology Auckland New Zealand

**Keywords:** ageing, foot, lower limb, older adult

## Abstract

**Background:**

Self‐perceptions of ageing have an important influence on the physical function in later life, yet little is known about how these perceptions relate to foot and lower limb health. Exploring how self‐perceptions of ageing interact with subjective reports of foot health as well as objective measures such as lower limb joint movement, muscle strength and functional mobility may provide important insights to support more person‐centred and responsive models of care. This study aimed to determine the association between self‐perceptions of ageing and subjective and objective measures of foot and lower limb health in older adults.

**Methods:**

This cross‐sectional study included 40 community‐dwelling adults ≥ 65 years who completed the five subscales of the Brief Ageing Perceptions Questionnaire (B‐APQ) alongside patient‐reported outcomes (100 mm Visual Analogue Scale [VAS] for foot pain, Manchester Foot Pain and Disability Index [MFPDI] and Lower Limb Task Questionnaire [LLTQ]). Objective assessments of joint range of motion, foot and ankle muscle strength and functional mobility tasks (timed up and go [TUG] and Short Physical Performance Battery [SPPB]) were also conducted. B‐APQ subscale scores were direction‐aligned so that higher scores reflect more negative ageing perceptions. Relationships between B‐APQ subscales and foot and lower limb outcomes were modelled using linear regression. All models were adjusted for key clinical and demographic confounders. Omnibus block tests evaluated the joint contribution of the five B‐APQ subscales.

**Results:**

The B‐APQ dimension block showed no evidence of association with foot pain (VAS, MFPDI). The B‐APQ block was associated with plantarflexion and inversion strength, with more adverse beliefs about consequences/low control (consequences‐control negative) and lower perceived control (control‐positive) associated with weaker strength. Consequences‐control negative was also associated with slower TUG, whereas SPPB total and joint motion showed no evidence of association.

**Conclusion:**

Associations between self‐perceptions of ageing and lower limb function and mobility appear dimension‐specific with beliefs about adverse consequences and perceived control most consistently related to neuromuscular strength and mobility, rather than pain. Interventions combining progressive strengthening with strategies addressing specific ageing‐belief dimensions, may support mobility in older adults.

## 1 Background

The understanding of well‐being and function of older adults has become increasingly important as the global population ages [1]. Older adults are typically defined as those aged 65 years and over [2]. The growing older adult population has heightened the focus on supporting psychological as well as physical well‐being in ageing research and clinical practice [3, 4]. Increasing rates of age‐related conditions, including chronic illness, cognitive decline and mental health concerns, underscore the need to better understand factors that influence quality of life (QoL) in older adults [4].

Psychological responses to ageing are often understood through self‐perceptions of ageing (SPA), which is a multidimensional construct reflecting individuals' beliefs, expectations and interpretations of their own ageing process [5, 6]. An individuals' beliefs and attitudes towards their own ageing process is a key determinant of physical health outcomes [7, 8, 9] and evidence suggests negative SPA has been linked to lower engagement in physical activity, poorer daily functioning, cognitive decline and a reduced will to live [7, 10, 11, 12, 13]. In contrast, positive SPA is associated with greater life satisfaction, well‐being, physical activity, functional performance and improved recovery following injuries such as falls [14, 15].

The lower limb, including the foot and ankle, plays a critical role in independent mobility, balance and the ability to perform activities of daily living [16]. Recent cohort‐based studies highlight the importance of lower limb measures such as balance, gait speed and chair stand performance, which are highly sensitive to age‐related change and strongly associated with functional capacity in older adults [16, 17]. Ageing is consistently accompanied by reduced range of motion (ROM), diminished muscle strength, altered gait patterns and foot‐specific structural and sensory changes that impair loading and postural control [18, 19, 20]. Foot pain is common in later life and is linked to poorer health‐related QoL, difficulty performing daily activities, impaired balance and gait, a higher risk of falls and may contribute to pain in other joints due to compensatory movement patterns [21, 22, 23]. As self‐perceptions of ageing can shape the way people perceive symptoms, their activity choices and engagement with management, they may influence both what people report (e.g., pain, function) and how they perform on objective tests. This study therefore aimed to determine the associations between self‐perceptions of ageing and both patient‐reported outcomes and objective measures of foot‐ and lower limb health and performance.

## 2 Methods

### 2.1 Study Design

Cross‐sectional observational study.

### 2.2 Participants

Due to the lack of published data directly examining the association between self‐perceptions of ageing and foot health, an a priori sample size calculation was conducted in G*Power based on a conventional approach using a medium effect size (*r* = 0.5), a significance level (*α*) of 0.05 and a statistical power of 90% (1—*β* = 0.90) [24]. This resulted in a required sample size of 40 participants which was considered sufficient to detect a meaningful association while minimising the risk of Type II error. Participants were included if they were community‐dwelling adults aged 65 years and older, able to walk independently for 4 m (with or without a walking aid), had sufficient cognitive capacity and possessed adequate English literacy to complete questionnaires. Exclusion criteria included lower‐limb surgery or fractures within the past 6 months, neurological conditions or physical conditions that impaired their ability to complete the required physical tasks. Participants were recruited through community advertising with respondent‐initiated contact and eligibility pre‐screening. Convenience sampling was used with the aim of including a range of ages and genders. Ethical approval was obtained from Auckland University of Technology Ethics Committee (25/355), and all participants provided written informed consent prior to data collection.

### 2.3 Data Collection

All data was collected by two podiatrists (EB and MB) during a single study visit at the AUT podiatry clinic. EB was responsible for the administration of the patient‐reported outcome measures included in the subjective assessments, whereas MB was responsible for collecting all objective assessment data. Data were recorded on a standardised clinical report form developed for this study to ensure consistent capture of demographic, clinical and outcome measure data during the study visit (see Supporting Information S2). Demographic and clinical information collected included age, sex, ethnicity, height and weight (used to calculate body mass index), comorbidities and current medications.

#### 2.3.1 Subjective Assessments

##### 2.3.1.1 Brief Ageing Perceptions Questionnaire (B‐APQ)

The B‐APQ is a condensed version of the 32‐item Ageing Perceptions Questionnaire, consisting of 17 items [7]. This questionnaire assesses five dimensions related to SPA: time‐line (chronic) (constant awareness of ageing), consequences (positive) (beliefs about the impacts of ageing), control (positive) (beliefs about how much control one has over aspects of ageing), consequences and control (negative) (beliefs about the impacts of ageing and beliefs about how much control one has over aspects of ageing) and emotional representations (emotional responses to ageing) [7]. Participants related their agreement with each item on a 5‐point scale: ‘strongly disagree’, ‘disagree’, ‘neither agree nor disagree’, ‘agree’ and ‘strongly agree’ [7]. To facilitate consistent interpretation across subscales, scores were direction‐aligned so that higher values represent more negative ageing perceptions for all dimensions. Consequently, consequences (positive) and control (positive) were reverse‐scored using the transformation (6‐ raw score), whereas timeline (chronic), emotional representations and consequences and control (negative) were already oriented in the negative direction.

##### 2.3.1.2 100 mm Visual Analog Scale (VAS)

Foot pain severity was assessed using a 100‐mm VAS anchored by ‘no pain’ at one end and ‘extreme pain’ at the other [25]. Participants marked the point that best represented their current level of foot pain, and scores were calculated by measuring the distance in millimetres from the ‘no pain’ anchor [25].

##### 2.3.1.3 The Manchester Foot Pain & Disability Index (MFPDI)

The MFPDI is a 19‐item questionnaire assessing foot pain and disability [26]. Each item is rated as ‘none of the time’ (0), ‘some of the time’ [1] or ‘on most/everyday(s)’ [2, 26]. Total scores were calculated, with higher scores indicating a greater degree of foot pain and disability.

##### 2.3.1.4 The Lower Limb Task Questionnaire (LLTQ)

The LLTQ assesses difficulty performing lower‐limb related tasks and consists of two subsections: activities of daily living and recreational activities, each containing 10 items [27]. Participants rated their difficulty in performing each task over the last 24 h on a 5‐point Likert scale; no difficulty [4], mild difficulty [3], moderate difficulty [2], severe difficulty [1] and unable (0) [27]. The total score for each category (activities of daily living and recreation) was calculated, with higher scores indicating a lower degree of difficulty.

#### 2.3.2 Objective Assessments

Objective assessments of foot and lower limb function were conducted using a range of validated outcome measures appropriate for older adults [28]. All tests were consistent with routine podiatric clinical practice [28].

##### 2.3.2.1 Joint Range of Motion (ROM)

Goniometric measurements of first metatarsophalangeal joint (1st MTPJ) and ankle joint dorsiflexion ROM were obtained due to the importance of these movements in facilitating forward progression during gait [29]. Standard goniometric techniques were used to maximise measurement reliability [30, 31]. A universal plastic goniometer (1° increments) was used for all measurements. Participants were in a standard seated position. For 1st MTPJ ROM, the fulcrum of the goniometer was placed over the medial 1st MTPJ with the stationary arm aligned with the medial midline of the first metatarsal and moving arm aligned with the medial midline of the proximal phalanx. The examiner passively dorsiflexed the hallux to a firm end‐feel while stabilising the first ray. For ankle dorsiflexion, the fulcrum of the goniometer was placed over the lateral malleolus with the stationary arm along the lateral midline of the fibular and the moving arm parallel to the lateral boarder of the fifth metatarsal. The examiner passively dorsiflexed the foot to a firm end‐feel while stabilising the lower leg. As ROM measurements can be influenced by participant positioning and assessor technique, three repeated measures were collected and averaged to reduce random measurement error and improve reliability. The three repeated trials demonstrated excellent within‐session examiner (MB) reliability for 1st MTPJ ROM (ICC (3, 3) = 0.997, 95% CI 0.996–0.998; SEM = 1.28) and ankle ROM (ICC(3, 3) = 0.997, 95% CI 0.996–0.998; SEM = 0.77).

##### 2.3.2.2 Foot and Ankle Muscle Strength

Foot and ankle muscle strength of plantarflexion, dorsiflexion, inversion and eversion were assessed using a fixed multi‐joint dynamometry force frame (VALD Health ForceFrame). Participants were tested in a standardised seated position with the knee and ankle positioned at 90° [32]. Each contraction was held for three seconds with 30 s rest between trials [32]. Participants were encouraged to exert as much force as they could for each contraction. Peak force (*N*) was recorded for all movement directions to provide objective measures of muscle strength. Fixed‐frame dynamometry has strong evidence supporting its validity in older adults, with high intra‐ and inter‐rater reliability for isometric ankle strength assessment when stabilisation and joint positions are standardised [32]. As muscle testing is effort‐dependent and influenced by familiarisation, the highest value from three trials was used to represent true maximal voluntary contraction [32]. Examiner (MB) reliability was excellent for plantarflexion strength (ICC(3, 1) = 0.94, 95% CI 0.915–0.959; SEM = 63.2), dorsiflexion strength (ICC(3, 1) = 0.942, 95% CI 0.918–0.961; SEM = 17.0), inversion strength (ICC(3, 1) = 0.943, 95% CI 0.918–0.961; SEM = 8.22) and eversion strength (ICC(3, 1) = 0.938, 95% CI 0.912–0.958; SEM = 9.65).

##### 2.3.2.3 Timed‐Up and Go Test

Functional mobility was assessed using the timed up and go (TUG) test. Participants were instructed to stand up from a standard chair, walk 3 m, turn around, return to the chair and sit down [33]. Each participant completed two timed trials, with the fastest time (in seconds) used for analysis. Performance was interpreted according to established thresholds for older adults: < 10 s indicates free mobility and low fall risk; 10–13.5 s indicates mostly independent mobility with moderate fall risk; and > 13.5 s reflects an increased fall risk [33].

##### 2.3.2.4 Short Physical Performance Battery

Lower limb function was also assessed using the Short Physical Performance Battery (SPPB), a composite measure that evaluates balance, gait speed and repeated chair stands [34]. Total scores range from 0 to 12, with higher scores indicating better physical performance [34].

Balance was measured using the three standard SPPB stances. Participants first attempted the side‐by‐side stance, standing with their feet together. This was scored as 1 if the position was held for 10 s and 0 if it was not held for 10 s or not attempted [34]. Participants then performed the semi‐tandem stance, placing one foot slightly in front of the other so that the medial hallux touched the medial aspect of the opposite calcaneus. This was scored as 1 if held for 10 s and 0 if not held for 10 s or not attempted [34]. Finally, participants attempted the tandem stance, standing with one foot directly in front of the other, such that the toes of the back foot touched the heel of the front foot. This was scored as 2 if held for 10 s, 1 if held for 3–9.99 s and 0 if held for less than 3 s or not attempted [34].

The second domain assessed was gait speed, measured over a 4‐m timed walk at the participants usual walking pace [34]. A 1‐m acceleration zone and a 1‐m deceleration zone were included, resulting in a total walking distance of 6 m [34]. Two trials were performed, and the faster time was used for scoring. Scores were assigned as follows: 0 = unable; 1 = ≥ 8.70 s; 2 = 6.21–8.70 s; 3 = 4.82–6.20 s; 4 = ≤ 4.82 s [34].

The third domain, lower limb strength and functional power were assessed using the repeated chair‐stand test [34]. Participants sat with their arms crossed over their chest and completed five consecutive sit‐to‐stand repetitions as quickly as possible. The total time in seconds was recorded [34]. Scoring followed established validated time thresholds ranging from 0 to 4: 0 = unable to complete one stand without using arms or unable to complete all five stands; 1 = ≥ 16.7 s, 2 = 13.7–16.69 s, 3 = 11.2–13.69 s, 4 = ≤ 11.19 s [34].

For the purpose of the analysis, individual domain scores were summated to produce a single total SPPB score which has established construct and prognostic validity, including predicting subsequent disability, falls and mortality [34, 35, 36].

## 3 Data Analysis

All analyses were performed in RStudio (version 2025.09.1). Continuous variables were summarised using mean (SD) and categorical variables as *N* (%). Associations between B‐APQ subscale scores and foot‐level outcomes (ROM and muscle strength) were examined using linear mixed‐effects models (*lme4*, *lmerTest*), including a random intercept for participant to account for repeated left/right foot measurements nested within participants. Associations between B‐APQ subscale scores and participant‐level outcomes (TUG, SPPB total score and patient‐reported outcomes) were examined using standard linear regression (*lm*). In all models, the five B‐APQ subscale scores were entered simultaneously as fixed effects (multivariable approach). All analyses were adjusted a priori for age (continuous), gender (categorical), number of comorbidities (continuous) and polypharmacy group (ordinal; reference category = no polypharmacy). For foot‐level outcomes, foot side was included as a fixed effect. An omnibus (block) test evaluated whether the set of B‐APQ subscales jointly explained variance in each outcome. Subscale‐specific inference was contingent on a significant block test; otherwise, subscale estimates are presented as hypothesis‐generating. For mixed‐effects models, this was performed using a likelihood ratio test comparing nested models that differed only by the inclusion of the B‐APQ subscale block (with covariates and foot side retained in both models). For standard linear regression models, an omnibus Wald test was used. Collinearity among B‐APQ subscales was assessed using variance inflation factors (VIFs). Model fit was summarised using marginal and conditional *R* ^2^ for mixed‐effects models and *R* ^2^/adjusted *R* ^2^ for standard regression models. Model assumptions were assessed via visual inspection of Pearson residuals (histograms and Q–Q plots for normality; residuals‐versus‐fitted and scale–location plots for homoscedasticity). Fixed‐effect coefficients (*β*), 95% Wald confidence intervals and *p*‐values were reported to describe subscale‐specific partial associations adjusted for the other subscales and covariates. Given the number of outcomes examined, patterns supported by significant omnibus block tests were emphasised and estimates from nonsignificant blocks were interpreted as exploratory.

## 4 Results

### 4.1 Participants

Participant characteristics including demographic information, comorbidities, medication use, polypharmacy categories and scores across the B‐APQ subscales of the 40‐community dwelling older adults aged 65 and over are presented in Table 1.

**TABLE 1:** Participant characteristics (*n* = 40).

Age (mean, SD) (years)		76.6 (7.0)
Gender (*n*, %)	Male	11 (27.5%)
	Female	29 (72.5%)
Ethnicity (*n*, %)	NZ European/Pakeha	33 (82.5%)
	Other European	7 (17.5%)
BMI (mean, SD)		27.9 (4.4)
Comorbidities (*n*, %)	Hypertension	27 (67.5%)
	Hyperlipidaemia	13 (32.5%)
	Diabetes mellitus	4 (10.0%)
	Osteoarthritis	12 (30.0%)
	Depression	9 (22.5%)
	Chronic kidney disease	1 (2.5%)
	Cardiovascular disease	4 (10.0%)
Medications (*n*, %)	Anti‐hypertensives	28 (70.0%)
	Lipid‐lowering agents	14 (35.0%)
	Analgesics	15 (37.5%)
	Anti‐inflammatories	14 (35.0%)
	Anti‐diabetics	5 (12.5%)
Polypharmacy (*n*, %)	No polypharmacy (0–4)	24 (60.0%)
	Polypharmacy (5–9)	11 (27.5%)
	Hyper‐polypharmacy (≥ 10)	5 (12.5%)
B‐APQ subscale scores (mean, SD)	Timelinechronic	2.7 (0.9)
	Consequences‐positive	2.1 (0.6)
	Emotional representations	2.2 (0.7)
	Consequences & control‐negative	2.8 (0.7)
	Control‐positive	1.7 (0.7)

*Note:* B‐APQ subscale scores are presented as direction‐aligned (reverse‐scored where applicable) such that higher scores indicate more negative ageing perceptions.

Abbreviations: B‐APQ: Brief‐Ageing Perception Questionnaire, BMI: Body Mass Index, SD: Standard Deviation.

### 4.2 Associations Between Self‐Perceptions of Ageing and Patient‐Reported Measures of Foot and Lower Limb Health

Descriptive statistics for patient‐reported measures of foot and lower limb health are shown in Table 2. Across patient‐reported outcomes, the B‐APQ subscale block showed no evidence of association with foot pain VAS or MFPDI (omnibus Wald tests nonsignificant; Table 3). Consistent with the analysis plan, subscale‐specific estimates are therefore considered hypothesis generating. Within this context, the timeline‐chronic subscale showed a negative partial association with the LLTQ daily activities score (*β* = −3.22, 95% CI –5.71 to −0.72, *p* = 0.013), indicating worse daily activities with more chronic/unchangeable ageing perceptions, after adjustment for the other subscales and covariates. For the LLTQ recreational activities domain, the B‐APQ block was not significant, and no B‐APQ subscale showed a statistically significant partial association. Number of comorbidities was associated with worse LLTQ daily activities and recreational activities scores (see Supporting Information S2 for full covariate estimates).

**TABLE 2:** Descriptive statistics for subjective measures of foot & lower limb health.

100 mm foot pain VAS, (mm) mean (SD)	15.5 (22.4)
Manchester foot pain and disability index, mean (SD)	3.7 (6.5)
Lower limb task Questionnaire, mean (SD)	
Daily living activities	35.4 (6.1)
Recreational activities	23.1 (10.7)

Abbreviations: SD: Standard Deviation, VAS: Visual Analog Scale.

**TABLE 3:** Associations between self‐perceptions of ageing and subjective measures of foot and lower limb health.

	BAPQ‐1 timeline‐chronic		BAPQ‐2 consequences‐positive		BAPQ‐3 emotional representations		BAPQ‐4 consequences‐control negative		BAPQ‐5 control‐positive		Omnibus block test
	*β* (95% CI)	*p*	*β* (95% CI)	*p*	*β* (95% CI)	*p*	*β* (95% CI)	*p*	*β* (95% CI)	*p*	
Foot pain VAS	4.36 (−9.03, 17.75)	0.511	−0.86 (−15.60, 13.88)	0.906	2.98 (−10.71, 16.67)	0.660	−10.02 (−28.70, 8.65)	0.281	−2.34 (−16.54, 11.86)	0.739	F (5, 29) = 0.29, *p* = 0.917, *R* ^2^/adj.*R* ^2^ = 0.11/−0.20
MFPDI	1.40 (−2.07, 4.86)	0.417	−1.38 (−5.20, 2.43)	0.464	0.52 (−3.02, 4.07)	0.766	−1.98 (−6.81, 2.86)	0.410	−0.72 (−4.40, 2.95)	0.690	F (5, 29) = 0.46, *p* = 0.800, *R* ^2^/adj.*R* ^2^ = 0.29/0.05
LLTQ (daily)	−3.22 (−5.71, −0.72)	0.013	−0.36 (−3.10, 2.39)	0.792	0.49 (−2.06, 3.04)	0.697	−0.86 (−4.34, 2.62)	0.617	−2.51 (−5.15, 0.14)	0.062	F (5, 29) = 2.20, *p* = 0.082, *R* ^2^/adj.*R* ^2^ = 0.58/0.43
LLTQ (recreation)	−4.41 (−9.07, 0.25)	0.062	−3.29 (−8.42, 1.84)	0.200	0.30 (−4.46, 5.06)	0.898	−3.58 (−10.07, 2.92)	0.270	−3.35 (−8.29, 1.59)	0.176	F (5, 29) = 1.89, *p* = 0.127, *R* ^2^/adj.*R* ^2^ = 0.53/0.36

*Note:* Omnibus (block) tests evaluate whether the five B‐APQ subscales jointly explain variance in each outcome. Bolded subscale *p*‐values indicate *p* < 0.05 only when the omnibus block test is significant (confirmatory inference). Where the omnibus test is nonsignificant (or borderline), subscale *p*‐values are presented as exploratory/hypothesis‐generating and are not bolded. All models were adjusted a priori for age, gender, number of comorbidities, and polypharmacy (see Supporting Information S1 for full model outputs). Positive *β* coefficients reflect higher PROM scores for each 1 point increase in B‐APQ subscale scores, where higher scores represent more negative ageing perceptions.

Abbreviations: B‐APQ: Brief‐Ageing Perception Questionnaire, CI: confidence interval, LLTQ: Lower Limb Task Questionnaire, MFPDI: Manchester Foot Pain and Disability Index, VAS: visual analog scale.

### 4.3 Associations Between Self‐Perceptions of Ageing and Objective Measures of Foot and Lower Limb Function

Descriptive statistics for objective measures of foot and lower limb function are shown in Table 4. Across outcomes, the B‐APQ subscale block did not explain variability in joint ROM (1^st^ MTPJ or ankle), with nonsignificant omnibus tests and no evidence of subscale‐specific partial associations (all *p* > 0.05) (Table 5). In contrast, for muscle strength, there was evidence that the subscales as a set were associated with plantarflexion and inversion strength (omnibus *χ* ^*2*^ [5] = 12.29, *p* = 0.031; and *χ* ^*2*^ [5] = 16.91, *p* = 0.005, respectively). In these models, higher (more adverse) scores on the consequences‐control negative and control‐positive subscales were associated with lower plantarflexion and inversion strength (negative partial associations) after mutual adjustment for other B‐APQ subscales and covariates. For eversion strength, the omnibus block test was borderline/nonsignificant (*χ* ^*2*^ [5] = 10.99, *p* = 0.052). Accordingly, although partial estimates for consequences‐control negative and control‐positive retained statistically significant negative partial associations (*β* = −22.65, *p* = 0.025; and *β* = −17.49, *p* = 0.023), these are interpreted as hypothesis‐generating rather than confirmatory. For dorsiflexion strength, there was no evidence of an association for the subscale block and no subscale‐specific partial associations. For global function, the subscale block was associated with TUG performance (F (5,29) = 2.62, *p* = 0.045), driven by a slower (worse) TUG time with higher consequences‐control negative scores (*β* = 1.43, 95% CI 0.42–2.44, *p* = 0.007). In contrast, SPPB total score showed no evidence of association with the subscale block. Several covariates showed independent associations with objective outcomes (full estimates in Supporting Information S1). Notably, age was associated with poorer plantarflexion, inversion and eversion strength and slower TUG time; male gender was associated with greater dorsiflexion, inversion and eversion strength; and hyper‐polypharmacy was associated with ankle ROM and 1st MTPJ ROM in some models.

**TABLE 4:** Descriptive statistics for objective measures of foot & lower limb function.

		Right foot	Left foot
1^st^ MTP dorsiflexion ROM (°), mean (SD)		79.5 (20.5)	78.2 (19.9)
Ankle dorsiflexion ROM (°), mean (SD)		14.9 (14.3)	14.0 (13.4)
Plantarflexion muscle strength (*N*), mean (SD)		683.0 (265.1)	726.3 (240.2)
Dorsiflexion muscle strength (*N*), mean (SD)		189.2 (67.3)	178.7 (71.5)
Inversion muscle strength (*N*), mean (SD)		70.2 (35.4)	66.3 (34.9)
Eversion muscle strength (*N*), mean (SD)		99.2 (40.3)	93.2 (39.3)
TUG time (sec), mean (SD)		8.8 (2.2)	
SPPB scores, mean (SD)	Balance	3.2 (1.0)	
	Gait speed	3.9 (0.4)	
	Chair stand	3.4 (1.0)	
	Total score	10.5 (1.8)	

Abbreviations: *N*: Newtons, ROM: Range of Motion, SD: Standard Deviation, SPPB: Short Physical Performance Battery, TUG: Timed up‐and‐go.

**TABLE 5:** Associations between self‐perception of ageing and objective measures of foot and lower limb function.

	B‐APQ timeline‐chronic		B‐APQ consequences‐positive		B‐APQ emotional representations		B‐APQ consequences‐control negative		B‐APQ control‐positive		Omnibus block test
	*β* (95% CI)	*p*	*β* (95% CI)	*p*	*β* (95% CI)	*p*	*β* (95% CI)	*p*	*β* (95% CI)	*p*	
1MTP ROM	2.71 (−5.69, 11.12)	0.518	−0.54 (−9.79, 8.71)	0.907	−0.06 (−8.65, 8.53)	0.989	−0.18 (−11.90, 11.54)	0.975	0.39 (−8.52, 9.30)	0.930	*χ* ^*2*^ (5) = 0.70, *p* = 0.983, R^2^m/R^2^c = 0.32/0.85
Ankle ROM	3.47 (−2.96, 9.90)	0.282	−1.76 (−8.84, 5.32)	0.618	0.17 (−6.41, 6.75)	0.959	−4.04 (−13.01, 4.93)	0.369	−1.12 (−7.94, 5.70)	0.742	*χ* ^*2*^ (5) = 2.46, *p* = 0.783, R^2^m/R^2^c = 0.22/0.96
DF strength	18.27 (−7.42, 43.97)	0.158	6.41 (−21.88, 34.70)	0.650	−3.36 (−29.63, 22.92)	0.798	−19.09 (−54.93, 16.75)	0.288	−18.15 (−45.40, 9.10)	0.186	*χ* ^*2*^ (5) = 6.02, *p* = 0.304, R^2^m/R^2^c = 0.40/0.75
PF strength	−18.55 (−105.85, 68.75)	0.670	18.61 (−77.50, 114.71)	0.698	−14.52 (−103.78, 74.75)	0.744	−131.42 (−253.18, −9.67)	**0.035**	−116.30 (−208.87, −23.74)	**0.015**	*χ* ^*2*^ (5) = 12.29, *p* = **0.031**, R^2^m/R^2^c = 0.51/0.86
Inversion strength	7.58 (−4.28, 19.45)	0.204	−3.62 (−16.68, 9.44)	0.579	5.60 (−6.53, 17.73)	0.356	−26.74 (−43.29, −10.20)	**0.002**	−18.99 (−31.56, −6.41)	**0.004**	*χ* ^*2*^ (5) = 16.91, *p* = **0.005**, R^2^m/R^2^c = 0.54/0.88
Eversion strength	4.89 (−9.21, 18.99)	0.487	3.41 (−12.11, 18.93)	0.659	−0.44 (−14.86, 13.97)	0.951	−22.65 (−42.32, −2.99)	0.025	−17.49 (−32.44, −2.55)	0.023	*χ* ^*2*^ (5) = 10.99, *p* = 0.052, R^2^m/R^2^c = 0.49/0.85
TUG	0.07 (−0.66, 0.80)	0.843	−0.08 (−0.88, 0.72)	0.845	−0.19 (−0.94, 0.55)	0.599	1.43 (0.42, 2.44)	**0.007**	0.34 (−0.43, 1.11)	0.370	F (5, 29) = 2.62, *p* = **0.045**, *R* ^2^/adj.*R* ^2^ = 0.72/0.63
SPPB total score	0.05 (−0.83, 0.93)	0.904	−0.06 (−1.03, 0.91)	0.896	−0.37 (−1.27, 0.53)	0.405	−0.46 (−1.68, 0.77)	0.455	0.05 (−0.88, 0.99)	0.908	F (5, 29) = 0.54, *p* = 0.744, *R* ^2^/adj.*R* ^2^ = 0.37/0.16

*Note:* Omnibus (block) tests evaluate whether the five B‐APQ subscales jointly explain variance in each outcome. Bolded subscale *p*‐values indicate *p* < 0.05 only when the omnibus block test is significant (confirmatory inference). Where the omnibus test is nonsignificant (or borderline), subscale *p*‐values are presented as exploratory/hypothesis‐generating and are not bolded. All models were adjusted a priori for age, gender, number of comorbidities, and polypharmacy; for foot‐level outcomes, foot side was additionally included as a fixed effect (see Supporting Information S1 for full model outputs). Positive *β* coefficients reflect higher ROM, strength, TUG times, and SPPB total scores for each 1‐point increase in B‐APQ subscale scores, where higher scores represent more negative ageing perceptions.

Abbreviations: 1MTP: first metatarsophalangeal joint, B‐APQ: Brief‐Ageing Perception Questionnaire, CI: confidence interval, DF: dorsiflexion, PF: plantarflexion, ROM: range of motion, SPPB: Short Physical Performance Battery, TUG: timed‐up‐and‐go test total score.

## 5 Discussion

In this cross‐sectional study of community‐dwelling older adults, associations between SPA and foot and lower‐limb outcomes were dimension‐specific. The clearest and most consistent pattern involved the consequences‐control negative dimension (beliefs about adverse consequences of ageing and limited control), which was associated with poorer objective performance (specifically weaker plantarflexion and inversion muscle strength and slower timed up and go times). The control‐positive dimension also showed negative partial associations with plantarflexion and inversion strength, suggesting that perceived control may be particularly relevant to effort‐dependent strength outcomes. In contrast, other dimensions (e.g., consequences‐positive and emotional representations) showed little evidence of association with most outcomes. Collectively, these findings suggest that certain components of SPA, especially perceived adverse consequences and low control, may be more closely tied to functional capability and mobility than to symptom severity. Given the cross‐sectional design, these associations should be interpreted as potentially bidirectional, whereby poorer strength and mobility may contribute to more negative beliefs about ageing consequences and control, whereas those beliefs may also reduce activity engagement and contribute to deconditioning.

For symptom‐based patient‐reported outcomes, there was no evidence that the B‐APQ dimensions as a set were associated with foot or lower‐limb pain. This may reflect an important distinction between experiencing pain and interpreting pain as a sign of problematic ageing. These results align with qualitative research which shows that many older adults consider themselves to be ageing successfully despite chronic pain, even while acknowledging pain as a potential barrier to activity [37]. Alternatively, older adults with chronic foot pain may have adapted over time, reducing the perceived relevance of foot pain to ageing beliefs.

For perceived functional difficulty (LLTQ), the B‐APQ set did not reach statistical significance and therefore subscale estimates should be interpreted as hypothesis‐generating. Within this context, the timeline‐chronic dimension (constant awareness of ageing) showed a negative partial association with LLTQ daily activities, suggesting that heightened chronic awareness of ageing may co‐occur with greater perceived difficulty in routine tasks. This is consistent with the idea that negative ageing stereotypes can shape behaviour by influencing health beliefs and practices [13]. Longitudinal research demonstrates that more negative self‐perceptions of ageing predict poorer functional health trajectories and declines in physical functioning over time [12, 38]. Proposed behavioural pathways include reduced engagement in health‐promoting behaviours such as physical activity and altered perceived control over health [8, 39]. Consequently, such perceptions can become self‐fulfilling, with downstream effects on both wellbeing and even longevity [12]. Many LLTQ items (e.g., ‘get out of bed in the morning’) involve routine tasks that remain unnoticed until difficulty emerges. As people age, increased effort in once‐automatic activities may be interpreted as markers of ageing, thereby reinforcing chronic awareness of ageing [13]. Evidence from studies of daily functioning suggests that perceived limitations are shaped not only by objective impairment but also by subjective appraisal processes, whereas greater awareness of age‐related losses is linked to poorer self‐rated health [40]. Clinically, this points to opportunities for person‐centred conversations that normalise effort in daily tasks and reframe ageing as modifiable. Such approaches may support engagement and self‐management [41, 42, 43, 44]. Notably, comorbidity count was independently related to worse LLTQ scores, underscoring that perceived difficulty reflects both health burden and ageing beliefs rather than pain alone.

Dimension‐specific associations were also most evidence for effort‐dependent objective outcomes. The B‐APQ dimensions jointly explained variance in plantarflexion and inversion muscle strength and in TUG performance with the most consistent association observed for the consequences‐control negative dimension (and for strength, also control‐positive). This pattern supports prior work where negative SPA has been associated with slower TUG performance but not to composite SPPB scores [45, 46] and supports the notion that ageing beliefs and behaviour‐linked may be more tightly coupled with behaviour‐linked capability than with passive joint mobility or aggregated indices.

Functionally, plantarflexion, inversion and eversion strength contribute to propulsion and frontal‐plane gait stability, and deficits in these muscle groups have been linked to slower gait speed and increased fall risk [18, 47, 48]. In the current study, evidence for eversion strength associations was less definitive because the subscale block test was borderline/nonsignificant. Any subscale‐specific eversion estimates should therefore be treated as exploratory. Mechanistically, beliefs about adverse consequences and low control may reduce activity confidence, foster cautious movement and contribute to deconditioning that erodes strength and mobility over time [13, 45, 46, 49, 50]. By contrast, joint ROM is influenced predominantly by joint and tissue properties (e.g., capsule stiffness, tendon elasticity, and degenerative change), which are less sensitive to SPA‐related behavioural influences, consistent with the lack of evidence for associations between B‐APQ dimensions and ROM in the current study. Similarly, the absence of an association with SPPB total score may reflect the composite nature of the SPPB, which may be less sensitive to the specific behavioural pathways captured by particular SPA dimensions.

Physiological stress pathways may also contribute. Negative SPA may operate as a chronic stressor, increasing systemic inflammatory/oxidative processes that impair muscle protein synthesis and physical capability [51, 52, 53, 54, 55]. Although causal inference is not possible here, the pattern of findings (associations concentrated in strength and TUG with null findings for ROM and SPPB) supports a focus on modifiable behavioural pathways. These may include progressive strengthening and graded activity, alongside interventions that address dimension‐specific beliefs, particularly perceived consequences and control. Age, sex and polypharmacy signals in the adjusted models further contextualise performance variation that co‐exists alongside SPA.

This study has a number of strengths and limitations. Firstly, the outcomes measures used mirrored routine podiatric practice, enhancing clinical validity and translation into everyday practice. Measurement procedures were standardised and demonstrated excellent reliability. SPA was examined against both patient‐reported outcomes and objective performance, providing a holistic view of beliefs, symptoms, perceived function and measured capacity. However, the cross‐sectional design precludes causal inference. The sample was relatively small (*n* = 40) and comprised high‐functioning, community‐dwelling older adults with low levels of foot pain and high SPPB scores. This raises the possibility of floor/ceiling effects and restricted variability, particularly for pain and SPPB. The sample was also predominantly European, limiting generalisability to other ethnic groups. A further limitation is the potential for inflated Type I error due to multiple testing across numerous outcomes. Although we used omnibus block tests to reduce over‐interpretation of individual B‐APQ dimensions, multiplicity across outcomes remains. Therefore, *p*‐values should be interpreted cautiously and findings (particularly those from outcomes with nonsignificant tests) should be considered as preliminary and hypothesis‐generating and may therefore reflect potential patterns that require confirmation in higher‐powered studies and models that reduce collinearity.

In conclusion, this study is the first to examine how domain‐specific dimensions of SPA relate to foot and lower limb health outcomes. Although there was no evidence of an association between the B‐APQ dimensions and symptom‐based measures such as foot pain, beliefs reflecting adverse consequences and low control were associated with weaker muscle strength and slower mobility. Targeting these specific belief dimensions alongside progressive strengthening and activity‐supportive care may help maintain or improve mobility and strength in older adults.

## Author Contributions

**Prue Molyneux:** conceptualization, funding acquisition, investigation, methodology, supervision, writing – original draft, writing – review and editing, project administration. **Ella Bloomfield:** investigation, writing – review and editing. **Miles Broderick:** investigation, writing – review and editing. **Sarah Stewart:** conceptualization, funding acquisition, methodology, formal analysis, supervision, writing – original draft, writing – review and editing.

## Funding

This study was funded by Auckland University of Technology Faculty of Health and Environmental Sciences Summer Student Scholarships awarded to E.B. and M.B. and also supported by funding through ASICS New Zealand. These organisations had no role in the study design, collection, analysis, interpretation of the data or in the decision to submit the article for publication.

## Conflicts of Interest

P.M. is on the editorial board for the Journal of Foot and Ankle Research. S.S., E.B. and M.B. declare no conflicts of interest in relation to this work.

## Supporting information


Supporting Information S1

Supporting Information S2

## Data Availability

The data that support the findings of this study are available from the corresponding author upon reasonable request.
