# Supplementary material for: The Association Between Self‐Perceptions of Ageing and Foot and Lower‐Limb Health in Community‐Dwelling Older Adults
Source: J Foot Ankle Res. 2026 Jun 8;19(2):e70172. doi: 10.1002/jfa2.70172 (PMC13246324; doi:10.1002/jfa2.70172)
Supplement: Supplementary file 1 — Supporting Information S1 [file JFA2-19-e70172-s001.docx]

**Supplementary File 1.** Results from fully-adjusted models

**Associations between self-perceptions of aging and subjective measures of foot and lower limb health**

| **Association between self-perceptions of aging and foot pain visual analog scale (VAS) score** | | |
| --- | --- | --- |
| **Term** | **β (95% CI)** | **P** |
| B-APQ-1 Timeline-Chronic | 4.36 (-9.03, 17.75) | 0.511 |
| B-APQ-2 Consequences-Positive | -0.86 (-15.60, 13.88) | 0.906 |
| B-APQ-3 Emotional representations | 2.98 (-10.71, 16.67) | 0.660 |
| B-APQ-4 Consequences-Control Negative | -10.02 (-28.70, 8.65) | 0.281 |
| BAPQ-5 Control-Positive | -2.34 (-16.54, 11.86) | 0.739 |
| Age (years) | 0.28 (-0.93, 1.49) | 0.637 |
| Gender (male vs female) | -1.10 (-21.25, 19.06) | 0.912 |
| Number of comorbidities | -0.40 (-7.19, 6.38) | 0.904 |
| Polypharmacy vs no polypharmacy | 8.33 (-13.61, 30.27) | 0.444 |
| Hyper-polypharmacy vs no polypharmacy | 19.06 (-14.13, 52.25) | 0.250 |
| **Omnibus block test** | | |
| F(5, 29)=0.29, p=0.917, R²/adj.R² = 0.11/-0.20 | | |

| **Association between self-perceptions of aging and Manchester Foot Pain and Disability Index (MFPDI) total score** | | |
| --- | --- | --- |
| **Term** | **β (95% CI)** | **P** |
| B-APQ-1 Timeline-Chronic | 1.40 (-2.07, 4.86) | 0.417 |
| B-APQ-2 Consequences-Positive | -1.38 (-5.20, 2.43) | 0.464 |
| B-APQ-3 Emotional representations | 0.52 (-3.02, 4.07) | 0.766 |
| B-APQ-4 Consequences-Control Negative | -1.98 (-6.81, 2.86) | 0.410 |
| BAPQ-5 Control-Positive | -0.72 (-4.40, 2.95) | 0.690 |
| Age (years) | 0.20 (-0.12, 0.51) | 0.212 |
| Gender (male vs female) | -0.46 (-5.67, 4.76) | 0.860 |
| Number of comorbidities | -0.33 (-2.08, 1.43) | 0.705 |
| Polypharmacy vs no polypharmacy | 3.29 (-2.39, 8.97) | 0.246 |
| Hyper-polypharmacy vs no polypharmacy | 8.05 (-0.55, 16.64) | 0.065 |
| **Omnibus block test** | | |
| F(5, 29)=0.46, p=0.800, R²/adj.R² = 0.29/0.05 | | |

| **Association between self-perceptions of aging and Lower Limb Task Questionnaire (LLTQ) – daily activities** | | |
| --- | --- | --- |
| **Term** | **β (95% CI)** | **P** |
| B-APQ-1 Timeline-Chronic | -3.22 (-5.71, -0.72) | **0.013** |
| B-APQ-2 Consequences-Positive | -0.36 (-3.10, 2.39) | 0.792 |
| B-APQ-3 Emotional representations | 0.49 (-2.06, 3.04) | 0.697 |
| B-APQ-4 Consequences-Control Negative | -0.86 (-4.34, 2.62) | 0.617 |
| BAPQ-5 Control-Positive | -2.51 (-5.15, 0.14) | 0.062 |
| Age (years) | -0.17 (-0.40, 0.05) | 0.129 |
| Gender (male vs female) | -1.24 (-4.99, 2.51) | 0.505 |
| Number of comorbidities | -1.68 (-2.94, -0.42) | **0.011** |
| Polypharmacy vs no polypharmacy | -0.44 (-4.53, 3.65) | 0.827 |
| Hyper-polypharmacy vs no polypharmacy | 0.51 (-5.68, 6.69) | 0.868 |
| Foot side (right vs left) |  |  |
| **Omnibus block test** | | |
| F(5, 29)=2.20, p=0.082, R²/adj.R² = 0.58/0.43 | | |

| **Association between self-perceptions of aging and Lower Limb Task Questionnaire (LLTQ) – recreational activities** | | |
| --- | --- | --- |
| **Term** | **β (95% CI)** | **P** |
| B-APQ-1 Timeline-Chronic | -4.41 (-9.07, 0.25) | 0.062 |
| B-APQ-2 Consequences-Positive | -3.29 (-8.42, 1.84) | 0.200 |
| B-APQ-3 Emotional representations | 0.30 (-4.46, 5.06) | 0.898 |
| B-APQ-4 Consequences-Control Negative | -3.58 (-10.07, 2.92) | 0.270 |
| BAPQ-5 Control-Positive | -3.35 (-8.29, 1.59) | 0.176 |
| Age (years) | -0.27 (-0.69, 0.15) | 0.200 |
| Gender (male vs female) | -0.07 (-7.08, 6.94) | 0.984 |
| Number of comorbidities | -3.43 (-5.79, -1.07) | **0.006** |
| Polypharmacy vs no polypharmacy | 3.45 (-4.18, 11.09) | 0.362 |
| Hyper-polypharmacy vs no polypharmacy | -0.08 (-11.63, 11.47) | 0.989 |
| **Omnibus block test** | | |
| F(5, 29)=1.89, p=0.127, R²/adj.R² = 0.53/0.36 | | |

**Associations between self-perception of aging and objective measures of foot and lower limb function**

| **Association between self-perceptions of aging and 1MTP dorsiflexion ROM** | | |
| --- | --- | --- |
| **Term** | **β (95% CI)** | **P** |
| B-APQ-1 Timeline-Chronic | 2.71 (-5.69, 11.12) | 0.518 |
| B-APQ-2 Consequences-Positive | -0.54 (-9.79, 8.71) | 0.907 |
| B-APQ-3 Emotional representations | -0.06 (-8.65, 8.53) | 0.989 |
| B-APQ-4 Consequences-Control Negative | -0.18 (-11.90, 11.54) | 0.975 |
| BAPQ-5 Control-Positive | 0.39 (-8.52, 9.30) | 0.930 |
| Age (years) | -0.70 (-1.45, 0.06) | 0.071 |
| Gender (male vs female) | -14.48 (-27.12, -1.83) | **0.026** |
| Number of comorbidities | 5.37 (1.12, 9.63) | **0.015** |
| Polypharmacy vs no polypharmacy | 6.78 (-7.95, 21.51) | 0.358 |
| Hyper-polypharmacy vs no polypharmacy | 13.48 (2.70, 24.26) | **0.016** |
| Foot side (right vs left) | -1.28 (-4.80, 2.23) | 0.464 |
| **Omnibus block test** | | |
| χ²(5)=0.70, p=0.983, R²m/R²c = 0.32/0.85 | | |

| **Association between self-perceptions of aging and ankle dorsiflexion ROM** | | |
| --- | --- | --- |
| **Term** | **β (95% CI)** | **P** |
| B-APQ-1 Timeline-Chronic | 3.47 (-2.96, 9.90) | 0.282 |
| B-APQ-2 Consequences-Positive | -1.76 (-8.84, 5.32) | 0.618 |
| B-APQ-3 Emotional representations | 0.17 (-6.41, 6.75) | 0.959 |
| B-APQ-4 Consequences-Control Negative | -4.04 (-13.01, 4.93) | 0.369 |
| BAPQ-5 Control-Positive | -1.12 (-7.94, 5.70) | 0.742 |
| Age (years) | 0.31 (-0.27, 0.89) | 0.290 |
| Gender (male vs female) | -3.81 (-13.49, 5.87) | 0.431 |
| Number of comorbidities | -2.47 (-5.73, 0.79) | 0.134 |
| Polypharmacy vs no polypharmacy | -1.27 (-12.54, 10.00) | 0.821 |
| Hyper-polypharmacy vs no polypharmacy | -10.92 (-19.17, -2.66) | **0.011** |
| Foot side (right vs left) | -0.93 (-2.25, 0.38) | 0.160 |
| **Omnibus block test** | | |
| χ²(5)=2.46, p=0.783, R²m/R²c = 0.22/0.96 | | |

| **Association between self-perceptions of aging and dorsiflexion muscle strength** | | |
| --- | --- | --- |
| **Term** | **β (95% CI)** | **P** |
| B-APQ-1 Timeline-Chronic | 18.27(-7.42,43.97) | 0.158 |
| B-APQ-2 Consequences-Positive | 6.41(-21.88,34.70) | 0.650 |
| B-APQ-3 Emotional representations | -3.36(-29.63,22.92) | 0.798 |
| B-APQ-4 Consequences-Control Negative | -19.09(-54.93,16.75) | 0.288 |
| BAPQ-5 Control-Positive | -18.15(-45.40,9.10) | 0.186 |
| Age (years) | -1.62(-3.93,0.70) | 0.167 |
| Gender (male vs female) | 67.25(28.57,105.93) | **0.001** |
| Number of comorbidities | -7.59(-20.61,5.43) | 0.246 |
| Polypharmacy vs no polypharmacy | -20.48(-65.52,24.56) | 0.364 |
| Hyper-polypharmacy vs no polypharmacy | 0.35(-32.63,33.33) | 0.983 |
| Foot side (right vs left) | -10.49(-26.15,5.17) | 0.183 |
| **Omnibus block test** | | |
| χ²(5)=6.02, p=0.304, R²m/R²c = 0.40/0.75 | | |

| **Association between self-perceptions of aging and plantarflexion muscle strength** | | |
| --- | --- | --- |
| **Term** | **β (95% CI)** | **P** |
| B-APQ-1 Timeline-Chronic | -18.55 (-105.85, 68.75) | 0.670 |
| B-APQ-2 Consequences-Positive | 18.61 (-77.50, 114.71) | 0.698 |
| B-APQ-3 Emotional representations | -14.52 (-103.78, 74.75) | 0.744 |
| B-APQ-4 Consequences-Control Negative | -131.42 (-253.18, -9.67) | **0.035** |
| BAPQ-5 Control-Positive | -116.30 (-208.87, -23.74) | **0.015** |
| Age (years) | -18.53 (-26.41, -10.66) | **<0.001** |
| Gender (male vs female) | 26.21 (-105.20, 157.61) | 0.689 |
| Number of comorbidities | -10.90 (-55.12, 33.33) | 0.621 |
| Polypharmacy vs no polypharmacy | 52.16 (-100.85, 205.18) | 0.495 |
| Hyper-polypharmacy vs no polypharmacy | 35.19 (-76.84, 147.22) | 0.529 |
| Foot side (right vs left) | 43.26 (-0.39, 86.90) | 0.052 |
| **Omnibus block test** | | |
| χ²(5)=12.29, p=0.031, R²m/R²c = 0.51/0.86 | | |

| **Association between self-perceptions of aging and inversion muscle strength** | | |
| --- | --- | --- |
| **Term** | **β (95% CI)** | **P** |
| B-APQ-1 Timeline-Chronic | 7.58 (-4.28, 19.45) | 0.204 |
| B-APQ-2 Consequences-Positive | -3.62 (-16.68, 9.44) | 0.579 |
| B-APQ-3 Emotional representations | 5.60 (-6.53, 17.73) | 0.356 |
| B-APQ-4 Consequences-Control Negative | -26.74 (-43.29, -10.20) | **0.002** |
| BAPQ-5 Control-Positive | -18.99 (-31.56, -6.41) | **0.004** |
| Age (years) | -1.47 (-2.54, -0.40) | **0.008** |
| Gender (male vs female) | 29.83 (11.98, 47.68) | **0.002** |
| Number of comorbidities | -5.81 (-11.82, 0.20) | 0.058 |
| Polypharmacy vs no polypharmacy | 9.68 (-11.11, 30.47) | 0.352 |
| Hyper-polypharmacy vs no polypharmacy | -2.36 (-17.58, 12.86) | 0.755 |
| Foot side (right vs left) | -3.94 (-9.44, 1.57) | 0.156 |
| **Omnibus block test** | | |
| χ²(5)=16.91, p=0.005, R²m/R²c = 0.54/0.88 | | |

| **Association between self-perceptions of aging and eversion muscle strength** | | |
| --- | --- | --- |
| **Term** | **β (95% CI)** | **P** |
| B-APQ-1 Timeline-Chronic | 4.89 (-9.21, 18.99) | 0.487 |
| B-APQ-2 Consequences-Positive | 3.41 (-12.11, 18.93) | 0.659 |
| B-APQ-3 Emotional representations | -0.44 (-14.86, 13.97) | 0.951 |
| B-APQ-4 Consequences-Control Negative | -22.65 (-42.32, -2.99) | **0.025** |
| BAPQ-5 Control-Positive | -17.49 (-32.44, -2.55) | **0.023** |
| Age (years) | -1.83 (-3.10, -0.56) | **0.006** |
| Gender (male vs female) | 29.34 (8.12, 50.56) | **0.008** |
| Number of comorbidities | -1.97 (-9.11, 5.17) | 0.581 |
| Polypharmacy vs no polypharmacy | -7.93 (-32.64, 16.78) | 0.520 |
| Hyper-polypharmacy vs no polypharmacy | -2.86 (-20.95, 15.23) | 0.751 |
| Foot side (right vs left) | -6.03 (-13.03, 0.97) | 0.089 |
| **Omnibus block test** | | |
| χ²(5)=10.99, p=0.052, R²m/R²c = 0.49/0.85 | | |

| **Association between self-perceptions of aging and Timed Up and Go (TUG) time** | | |
| --- | --- | --- |
| **Term** | **β (95% CI)** | **P** |
| B-APQ-1 Timeline-Chronic | 0.07 (-0.66, 0.80) | 0.843 |
| B-APQ-2 Consequences-Positive | -0.08 (-0.88, 0.72) | 0.845 |
| B-APQ-3 Emotional representations | -0.19 (-0.94, 0.55) | 0.599 |
| B-APQ-4 Consequences-Control Negative | 1.43 (0.42, 2.44) | **0.007** |
| BAPQ-5 Control-Positive | 0.34 (-0.43, 1.11) | 0.370 |
| Age (years) | 0.15 (0.08, 0.21) | 0.000 |
| Gender (male vs female) | -0.30 (-1.39, 0.80) | 0.581 |
| Number of comorbidities | 0.05 (-0.32, 0.41) | 0.805 |
| Polypharmacy vs no polypharmacy | 1.40 (0.21, 2.59) | **0.023** |
| Hyper-polypharmacy vs no polypharmacy | 1.37 (-0.43, 3.17) | 0.131 |
| **Omnibus block test** | | |
| F(5, 29)=2.62, p=0.045, R²/adj.R² = 0.72/0.63 | | |

| **Association between self-perceptions of aging and SPPB total score** | | |
| --- | --- | --- |
| **Term** | **β (95% CI)** | **P** |
| B-APQ-1 Timeline-Chronic | 0.05 (-0.83, 0.93) | 0.904 |
| B-APQ-2 Consequences-Positive | -0.06 (-1.03, 0.91) | 0.896 |
| B-APQ-3 Emotional representations | -0.37 (-1.27, 0.53) | 0.405 |
| B-APQ-4 Consequences-Control Negative | -0.46 (-1.68, 0.77) | 0.455 |
| BAPQ-5 Control-Positive | 0.05 (-0.88, 0.99) | 0.908 |
| Age (years) | -0.07 (-0.15, 0.01) | 0.094 |
| Gender (male vs female) | -0.16 (-1.49, 1.16) | 0.805 |
| Number of comorbidities | -0.13 (-0.58, 0.32) | 0.555 |
| Polypharmacy vs no polypharmacy | -1.32 (-2.76, 0.13) | 0.072 |
| Hyper-polypharmacy vs no polypharmacy | -0.76 (-2.95, 1.42) | 0.481 |
| **Omnibus block test** | | |
| F(5, 29)=0.54, p=0.744, R²/adj.R² = 0.37/0.16 | | |
