# Supplementary material for: The Association Between Self‐Perceptions of Ageing and Foot and Lower‐Limb Health in Community‐Dwelling Older Adults
Source: J Foot Ankle Res. 2026 Jun 8;19(2):e70172. doi: 10.1002/jfa2.70172 (PMC13246324; doi:10.1002/jfa2.70172)
Supplement: Supplementary file 2 — Supporting Information S2 [file JFA2-19-e70172-s002.pdf]

Clinical Report Form

Date of visit: ☐☐/☐☐/☐☐ PID: ☐☐☐

Demographic details *(student researchers to complete)*

Age

☐☐ years

Gender

☐ Female

☐ Male

☐ Gender diverse

☐ Prefer to self-describe: ________

Weight ☐☐☐ kg

Height ☐.☐☐ meters

Ethnicity

☐ Māori Iwi/hapu: ________

☐ Tokelauan

☐ Fijian

☐ Niuean

☐ Tongan

☐ Cook Islands Māori

☐ Samoan

☐ Other Pacific Peoples

☐ Southeast Asian

☐ Indian

☐ Chinese

☐ Other Asian

☐ Middle Eastern

☐ Latin American

☐ African

☐ Other Ethnicity

☐ Other European

☐ NZ European/Pākehā

Comorbidities (list)

Medications (list)

Patient Reported Outcome Measures *(participant to complete)*

Please indicate how much **foot pain** you have had in the **past week** by marking an **X** through the line:

No Pain ———————————————————— Extreme Pain

Please indicate below all the areas that you have experienced **foot pain** in the **past week**:

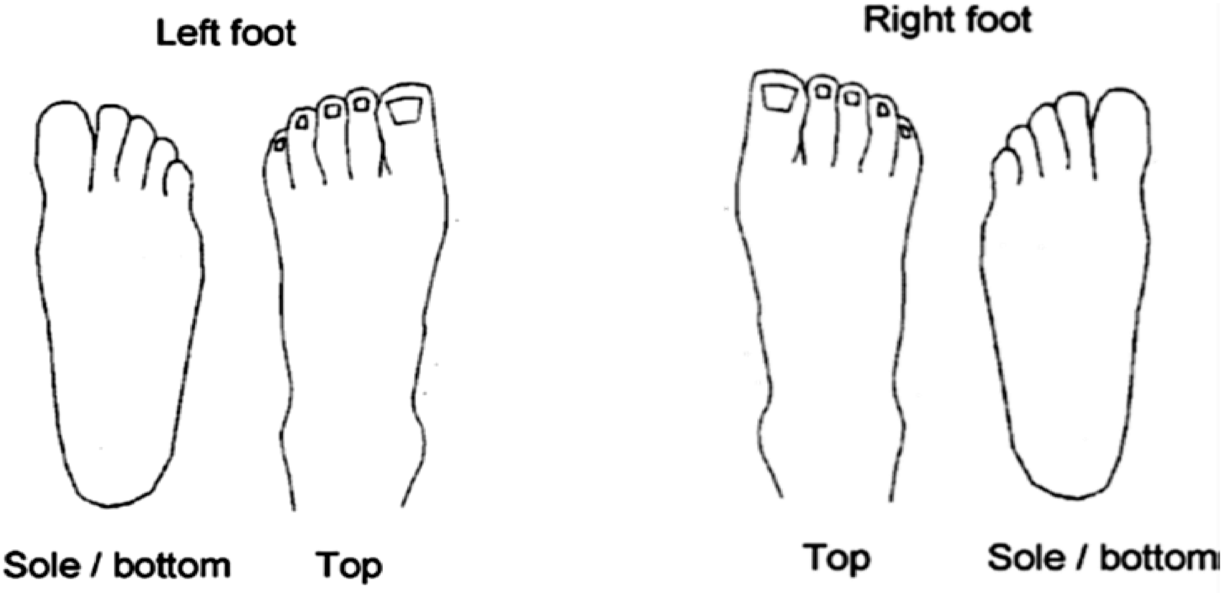

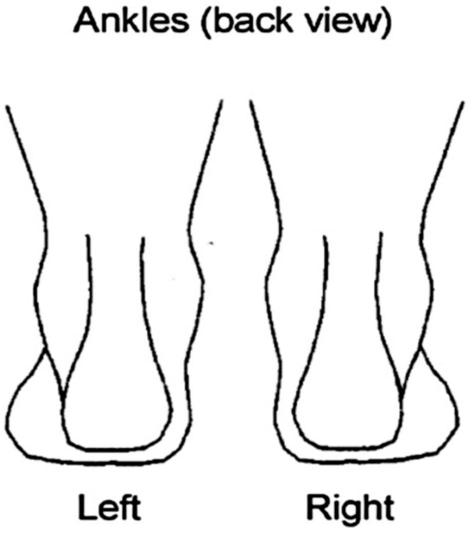

PID: ☐☐☐

Manchester Foot Pain and Disability Index

Below are some statements about problems people have because of pain in their feet.
For each statement, indicate if this has applied to you during the past month. If so, was this only on some days or on most or every day in the past month?

PLEASE TICK ONE BOX FOR EACH STATEMENT

	During the past month this has applied to me:			
	None of the time	On some days	On most/ everyday(s)	
**Because of pain in my feet:**				
I avoid walking outside at all	☐	☐	☐	
I avoid walking long distances	☐	☐	☐	
I don't walk in a normal way	☐	☐	☐	
I walk slowly	☐	☐	☐	
I have to stop and rest my feet	☐	☐	☐	
I avoid hard or rough surfaces when possible	☐	☐	☐	
**Because of pain in my feet:**				
I avoid standing for a long time	☐	☐	☐	
I catch the bus or use the car more often	☐	☐	☐	
I need help with housework/shopping	☐	☐	☐	
I still do everything but with more pain or discomfort	☐	☐	☐	
I get irritable when my feet hurt	☐	☐	☐	
I feel self-conscious about my feet	☐	☐	☐	
I get self-conscious about the shoes I have to wear	☐	☐	☐	
I have constant pain in my feet	☐	☐	☐	
My feet are worse in the morning	☐	☐	☐	
My feet are more painful in the evening	☐	☐	☐	
I get shooting pains in my feet	☐	☐	☐	
**Because of pain in my feet:**				Not applicable
I am unable to carry out my previous work	☐	☐	☐	☐
I no longer do all my previous activities (sport, dancing, hill-walking etc)	☐	☐	☐	☐

PID: ☐☐☐

Lower Limb Task Questionnaire
Activities of Daily Living Section

Please rate your ability to do the following activities in the **past 24 hours** by **circling** the number below the appropriate response.

If you did not have the opportunity to perform an activity in the past 24 hours, please make your best estimate on which response would be the most accurate.

	**Unable**	**Severe Difficulty**	**Moderate Difficulty**	**Mild Difficulty**	**No difficulty**
1. Walk for 10 minutes	0	1	2	3	4
2. Walk up or down 10 steps (1 flight)	0	1	2	3	4
3. Stand for 10 minutes	0	1	2	3	4
4. Stand for a typical work day	0	1	2	3	4
5. Get on and off a bus	0	1	2	3	4
6. Get up from a lounge chair	0	1	2	3	4
7. Push or pull a heavy shopping trolley	0	1	2	3	4
8. Get in and out of a car	0	1	2	3	4
9. Get out of bed in the morning	0	1	2	3	4
10. Walk across a slope/uneven ground	0	1	2	3	4

PID: ☐☐☐

Lower Limb Task Questionnaire

Recreational Activities Section

Please rate your ability to do the following activities in the **past 24 hours** by **circling** the number below the appropriate response.

If you did not have the opportunity to perform an activity in the past 24 hours, please make your best estimate on which response would be the most accurate.

	Unable	Severe Difficulty	Moderate Difficulty	Mild Difficulty	No difficulty
1. Jog for 10 minutes	0	1	2	3	4
2. Pivot or twist quickly while walking	0	1	2	3	4
3. Jump for distance	0	1	2	3	4
4. Run fast / sprint	0	1	2	3	4
5. Stop and start moving quickly	0	1	2	3	4
6. Jump upwards and land	0	1	2	3	4
7. Kick a ball hard	0	1	2	3	4
8. Pivot or twist quickly while running	0	1	2	3	4
9. Kneel on both knees for 5 minutes	0	1	2	3	4
10. Squat to the ground / floor	0	1	2	3	4

PID: ☐☐☐

Brief-Aging Perception Questionnaire

PLEASE TICK ONE BOX PER LINE WHICH SHOWS HOW YOU FEEL ABOUT EACH STATEMENT		STRONGLY DISAGREE	DISAGREE	NEITHER AGREE NOR DISAGREE	AGREE	STRONGLY AGREE
1	I always classify myself as old	☐	☐	☐	☐	☐
2	I am always aware of the fact that I am getting older	☐	☐	☐	☐	☐
3	I feel my age in everything that I do	☐	☐	☐	☐	☐
4	As I get older I get wiser	☐	☐	☐	☐	☐
5	As I get older I continue to grow as a person	☐	☐	☐	☐	☐
6	As I get older I appreciate things more	☐	☐	☐	☐	☐
7	I get depressed when I think about how ageing might affect the things that I can do	☐	☐	☐	☐	☐
8	The quality of my social life in later years depends on me	☐	☐	☐	☐	☐
9	The quality of my relationships with others in later life depends on me	☐	☐	☐	☐	☐
10	Whether I continue living life to the full depends on me	☐	☐	☐	☐	☐
11	Getting older makes me less independent	☐	☐	☐	☐	☐
12	As I get older I can take part in fewer activities	☐	☐	☐	☐	☐
13	As I get older I do not cope as well with problems that arise	☐	☐	☐	☐	☐
14	Slowing down with age is not something I can control	☐	☐	☐	☐	☐
15	I have no control over the effects which getting older has on my social life	☐	☐	☐	☐	☐

PID: ☐☐☐

16	I worry about the effects that getting older may have on my relationships with others	☐	☐	☐	☐	☐
17	I feel angry when I think about getting older	☐	☐	☐	☐	☐

PID: ☐☐☐

Objective assessments *(student researchers to complete)*

Joint range of motion testing

1MTP dorsiflexion ROM

	Right	Left
Trial 1		
Trial 2		
Trial 3		
Average		
Max		

Ankle dorsiflexion ROM

	Right	Left
Trial 1		
Trial 2		
Trial 3		
Average		
Max		

Muscle strength testing

Plantarflexion strength

	Right	Left
Trial 1		
Trial 2		
Trial 3		
Average		
Max		

Dorsiflexion strength

	Right	Left
Trial 1		
Trial 2		
Trial 3		
Average		
Max		

Inversion strength

	Right	Left
Trial 1		
Trial 2		
Trial 3		
Average		
Max		

Eversion strength

	Right	Left
Trial 1		
Trial 2		
Trial 3		
Average		
Max		

PID: ☐☐☐

Timed-Up-and-Go (TUG) Test

Refer to TUG protocol for instructions

Repeat two trials and record the time in seconds for each trial (we will use the best of both trials for the analysis)

Trial 1 [ ] seconds

Trial 2 [ ] seconds

Short Physical Performance Battery (SPPB) Test

Refer to SPPB protocol for instructions

1. Balance tests

Repeat one trial for each balance test and record the time in seconds for each test.

Side-by-side stand [ ] seconds

Semi-tandem [ ] seconds

Tandem [ ] seconds

Overall score: [ ]

0 = Unable to hold side-by-side for 10 sec; 1 = Holds side-by-side for 10 sec but not semi-tandem; 2 = Holds semi-tandem for 10 sec but not tandem; 3 = Holds tandem for 3–9.99 sec; 4 = Holds tandem for 10 sec

2. Gait speed test

Repeat two trials and record the time in seconds for each trial.

Trial 1 [ ] seconds

Trial 2 [ ] seconds

Overall score: [ ]

Based on the fastest of the two walking trials: 0 = Unable; 1 = ≥8.70 sec; 2 = 6.21–8.70 sec; 3 = 4.82–6.20 sec; 4 = ≤4.82 sec

3. Chair Stant Test

Repeat one trial and record the time taken for the participant to stand up 5 times.

Chair stand [ ] seconds

Overall score: [ ]

0 = Unable or >60 sec; 1 = ≥16.7 sec; 2 = 13.7–16.6 sec; 3 = 11.2–13.6 sec; 4 = ≤11.1 sec

Total SPPB score: [ ]

Add scores from all 3 components (range: 0–12)

PID: [ ] [ ] [ ]
